# Health Information Technology Usability Evaluation Scale (Health-ITUES) for Usability Assessment of Mobile Health Technology: Validation Study

**DOI:** 10.2196/mhealth.8851

**Published:** 2018-01-05

**Authors:** Rebecca Schnall, Hwayoung Cho, Jianfang Liu

**Affiliations:** ^1^ School of Nursing Columbia University New York, NY United States

**Keywords:** mobile technology, usability, mobile health apps, psychometric evaluation

## Abstract

**Background:**

Mobile technology has become a ubiquitous technology and can be particularly useful in the delivery of health interventions. This technology can allow us to deliver interventions to scale, cover broad geographic areas, and deliver technologies in highly tailored ways based on the preferences or characteristics of users. The broad use of mobile technologies supports the need for usability assessments of these tools. Although there have been a number of usability assessment instruments developed, none have been validated for use with mobile technologies.

**Objective:**

The goal of this work was to validate the Health Information Technology Usability Evaluation Scale (Health-ITUES), a customizable usability assessment instrument in a sample of community-dwelling adults who were testing the use of a new mobile health (mHealth) technology.

**Methods:**

A sample of 92 community-dwelling adults living with HIV used a new mobile app for symptom self-management and completed the Health-ITUES to assess the usability of the app. They also completed the Post-Study System Usability Questionnaire (PSSUQ), a widely used and well-validated usability assessment tool. Correlations between these scales and each of the subscales were assessed.

**Results:**

The subscales of the Health-ITUES showed high internal consistency reliability (Cronbach alpha=.85-.92). Each of the Health-ITUES subscales and the overall scale was moderately to strongly correlated with the PSSUQ scales (r=.46-.70), demonstrating the criterion validity of the Health-ITUES.

**Conclusions:**

The Health-ITUES has demonstrated reliability and validity for use in assessing the usability of mHealth technologies in community-dwelling adults living with a chronic illness.

## Introduction

Mobile technology has become a nearly ubiquitous technology in the United States, where almost two-thirds of the population owns a mobile phone, but also worldwide with more than half of the world population owning a mobile phone [1]. Mobile technology has the advantage of accessibility anywhere Internet access is available, relative affordability, and has been promoted specifically as a solution to reach stigmatized and disenfranchised populations [2,3]. Mobile technology can be particularly useful in the delivery of health interventions because this technology can allow us to deliver interventions to scale, cover broad geographic areas, and deliver technologies in highly tailored ways based on the preferences or characteristics of users [4,5].

Concurrent with the proliferation of mobile technology has been the vast increase in the number of mobile health (mHealth) apps available to consumers. For example, in 2013, a report by the IMS Institute for Healthcare Informatics estimated more than 40,000 health-related iPhone apps were available for consumer use [6]. By 2015, this number had more than doubled to 90,088 apps available on the iOS. In total, IMS estimated that 165,000 health apps were available for use both among iOS and Android platforms [6]. The mHealth apps use mobile devices (eg, mobile phones or tablets) for the deployment of health interventions and vary widely in the types of technology used (eg, text message reminders, app-based alerts and activities, and Web-based educational modules) [7].

The dramatic growth of mHealth technology is not surprising given that mobile technologies are a novel method for the delivery of cost-effective, timely, and relevant health promotion and management information [8]. Mobile delivery specifically has a number of key advantages over traditional face-to-face delivery models of care, including consumer control, decreased time burden, reduction of monetary and time costs associated with travel to a provider, and the ability to monitor and assess the use of digital analytics [8]. Additionally, mHealth technology presents an opportunity for consumers to self-monitor their health status and allows health care providers to organically reach persons who may be disengaged from the health care system [9]. Finally, mHealth technology allows for the dissemination of information quickly and broadly [10].

Use of mobile technology as a platform for behavioral interventions adjuncts to health care delivery and monitoring of health status has widely proliferated in the past 5 years. Despite this growth, few mHealth apps or interventions have undergone systematic and rigorous usability evaluation prior to their dissemination [11,12]. Usability evaluations remain challenging because of the cost and time necessary for completion of these assessments [13].

Another barrier to usability assessments is that a dearth of validated instruments for assessing mHealth technology persists [14-16]. Implications of insufficient usability testing in mHealth include the assertion that attrition is common within randomized controlled trials using technology due to usability errors [11,12,17]. However, without a standardized measure of usability, it is very difficult to assess, report on, and compare usability, as well as link it to attrition from the trial. At the same time, a standardized usability instrument would improve methodological consistency, making it possible to begin comparing findings across mHealth app evaluations [18].

In our earlier work, we presented preliminary validation of the Health Information Technology Usability Evaluation Model (Health-ITUEM), a theoretical framework to guide usability evaluations for assessing mHealth technologies [19]. The model was developed in response to the current gaps in the extant usability literature [20]. The Health-ITUEM is an integrated model of multiple usability theories based on the concepts of usability from the Technology Acceptance Model [21] and the International Organization for Standardization (ISO) standard 9241-11 [22]. The Health-ITUEM has been widely used as a framework for understanding the use of mobile technology studies since its validation [23,24], but there remains a lack of validated instruments for usability assessments of mHealth technology.

A need for a validated usability instrument for mHealth technologies persists. In response to this need, we sought to validate the use of the Health Information Technology Usability Evaluation Scale (Health-ITUES) in a sample of users of a mHealth app developed for community-dwelling adults living with HIV. The Health-ITUES, derived from the Health-ITUEM, is a validated instrument that explicitly considers task by addressing various levels of expectation of support for the task by the health information technology (IT). The Health-ITUES also has the added benefit of being customizable, which can address the study needs and concepts measured without item addition, deletion, or modification. This is an important benefit of this instrument because it allows for harmonization of findings across studies. The factorial validity and internal consistency of the Health-ITUES was demonstrated through an exploratory factor analysis [25]. The development of the Health-ITUES was further advanced through a confirmatory factor analysis and structural equation modeling to demonstrate its construct validity and predictive validity [26]. Early development and valuation of the Health-ITUES was conducted using a Web-based communication system that supported nurse staffing and scheduling. The Health-ITUES is not widely used and it has not been validated in a sample of patients or with the use of mobile technology. These two distinctions make the findings from this work highly relevant, timely, and provide an important contribution to the literature.

### Setting

These data were collected as part of a larger study for developing and testing a mobile app (mVIP) for symptom self-management in persons living with HIV. The goal of the parent study was to translate a paper-based manual that had been effective in ameliorating symptoms in persons living with HIV onto a mobile platform to promote dissemination of patient-centered outcomes research. As part of the parent study, we developed a beta version of the app and conducted end-user usability testing with 20 participants in a laboratory setting. Following refinement of the app based on our findings during our usability assessment, we tested the mobile app in a 12-week feasibility trial with 80 end users. Participants were randomized to an intervention or control group, but both groups received a version of the mobile app. The intervention group received an enhanced app, which included self-care strategies for 13 symptoms commonly experienced by persons living with HIV.

## Methods

Study participants were recruited from June 2016 to February 2017 through in-person and email recruitment. Recruitment sites included HIV clinics and community-based organizations. Eligibility criteria included: (1) diagnosed with HIV, (2) older than 18 years of age, (3) able to communicate in English, (4) experienced at least two of 13 HIV-related symptoms in the past week, (5) met the cognitive state minimum score (ie, 24 of 30) measured by the Mini-Mental State Examination (MMSE) [27], and (6) own a mobile phone or tablet. All participants completed written informed consent prior to the start of study activities. Following enrollment, study participants were given access to the mVIP app. A total of 20 participants enrolled as part of usability testing of the app and 80 participants enrolled in the 12-week feasibility study of the app. Data reported in this paper were collected at the baseline visit for each participant after he or she had used the app for the first time.

Participants in both groups were given access to the app on a mobile phone. Following log-in and use of the mobile app, participants completed a number of surveys, which are detailed subsequently. The surveys collected demographic information, the Health-ITUES [26], and the Post-Study System Usability Questionnaire (PSSUQ) [28]. Participants were given US $40 as a token of appreciation for their time for participating in the usability testing. Participants in the 12-week feasibility trial were given US $30 at baseline and US $40 at follow-up as a token of appreciation for their time.

### Instruments

#### Health Information Technology Usability Evaluation Scale

**Figure 1:**
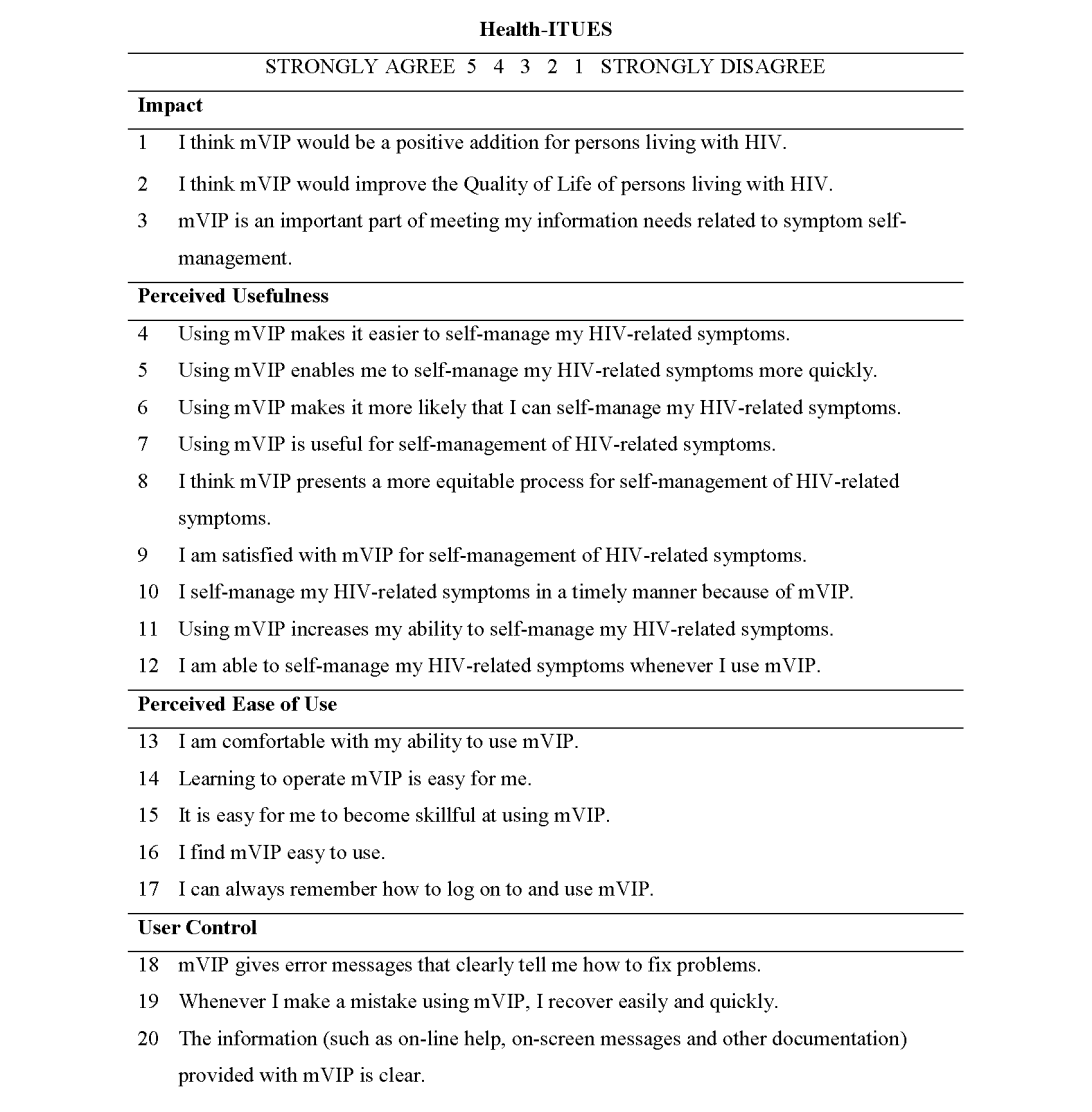
Untitled.

The Health-ITUES is a customizable questionnaire with a four-factor structure. The Health-ITUES explicitly considers a task by addressing various levels of expectation of support for the task by the health IT system, and it has been validated through exploratory factor analysis and confirmatory factor analysis in a sample of nurses who used a Web-based nurse-scheduling tool. The Health-ITUES consists of 20 items rated on a five-point Likert scale from strongly disagree (1) to strongly agree (5). A higher scale value indicates higher perceived usability of the technology. Items in each of these scales and how they were customized for this study are illustrated in Figure 1. The 20-item scale is comprised of four subscales: (1) quality of work life, (2) perceived usefulness, (3) perceived ease of use, and (4) user control. User control and perceived ease of use capture user-system interaction, whereas perceived usefulness evaluates task accomplishment through system use and quality of work life represents higher expectations of system impact. In the earlier studies, quality of work life referred to the system impact on work life; in our study with community-dwelling persons living with HIV, quality of work life represents the system impact on daily life. As a result, we renamed this factor structure for purposes of this validation as “impact.” The overall Health-ITUES score was the mean of all the items with each item weighted equally.

#### Sociodemographic Questionnaire

A self-reported sociodemographic questionnaire was developed to collect information on the participants’ age, gender, race, ethnicity, sexual orientation, education, annual income, relationship status, and a previous acquired immune deficiency syndrome (AIDS) diagnosis. Participants were also asked about their technology use with questions specifically asking about most frequently used type of mobile device, frequency of use of this device, and whether they use apps on their mobile phone.

#### Post-Study System Usability Questionnaire

The PSSUQ is an instrument for assessing user satisfaction with system usability and was developed as a usability assessment tool that was specifically for use in the context of scenario-based usability testing, although additional research has indicated that this may be useful for field evaluation as well [29,30]. Factor analysis of PSSUQ support a three-factor structure: system usefulness, information quality, and interface quality. For this study, we used the PSSUQ version 3, which is comprised of 16 items [28]. The items are rated on a seven-point scale, anchored at the end points with the terms strongly agree (1), strongly disagree (7), and a “not applicable” (N/A) point outside the scale [28,31]. The overall PSSUQ and subscale scores were reversely coded in this study so that higher scores indicated better user satisfaction. As a follow-up to the original PSSUQ, the developers of the instrument collected data from 5 years of usability studies and found similar psychometric properties between the original and the follow-up PSSUQ data despite the passage of time and differences in the types of systems studied, providing evidence of significant generalizability of the instrument for measuring participant satisfaction with the usability of tested systems [28].

### Procedures

Descriptive statistics were used to calculate demographic characteristics of the study sample. We used the Kruskal-Wallis test to determine if there was a relationship between any of the demographic data and each of the Health-ITUES constructs: impact, perceived usefulness, perceived ease of use, and user control.

Psychometric test theory involves the development and evaluation of clusters of questions called scales, which are used to gather information about the quality of psychological measures [32]. The Health-ITUES has been previously tested using exploratory factor analysis, confirmatory factor analysis, and structural equation modeling, as described previously, but it had not been validated in patients or with mobile technology. Therefore, there is a need for a further psychometric evaluation of this instrument. We evaluated the following properties in our study sample: variability, internal consistency reliability, construct validity, and criterion validity. Each property and the appropriate analysis are described subsequently. Outliers were checked and removed in the reliability and validity analysis.

Variability refers to the extent to which the full range of scale scores and item responses are reported in the data. Optimal variability is denoted by a full range of responses of the scale. Scales that are skewed, either negatively or positively, tend to be less responsive to the effect of usability errors. To ensure limits in the variability, the frequency of missing data needs to be limited and randomly distributed across participant responses. To address skewness, we used a nonparametric method (Kruskal-Wallis) to test the known-group validity. For the other analyses, such as Cronbach alpha and Pearson correlation, normality is not an assumption, so no log transformation was performed in this analysis.

Internal consistency reliability is a measure of the how well the instrument measures different constructs and delivers reliable scores. Internal consistency reliability measures whether several items that propose to measure the same general construct produce similar scores, indicating the homogeneity of a scale or subscale. The Cronbach alpha coefficient provides an estimated score of the internal consistency reliability based on all possible correlations between items collected at any time point [33]. Cronbach alpha scores range from .0 (no reliability) to 1.0 (perfect reliability) with the desired range of scores between .70 and .95 [34]. Cronbach alphas greater than .95 demonstrate highly correlated items, which is not desired.

Validity refers to how well the scale measures the attribute it claims to measure [35,36]. There are several components to validity; in this study, we focused on the construct and criterion validity. We measured three subtypes of construct validity in our study: convergent validity, discriminant validity, and known-group validity. Convergent validity refers to the degree to which theoretically correlated measures are in fact correlated, whereas discriminant validity is used to evaluate the differences between uncorrelated and correlated subscales [37]. One way to examine the convergent and discriminant validity is to assess the correlations among scale scores within the instrument based on known relationships. For example, scales measuring perceived ease of use are expected to correlate moderately with one another, whereas scales measuring impact are expected to have weaker correlations with user control because they measure different constructs. We used a multitrait-multimethod matrix [38] with interscale correlations to assess the convergent and discriminant validity. We also assessed the known-groups validity, which tests for anticipated differences on specific scale scores between groups that are known to be different [39]. In the case of our study population, we evaluated the ability of this instrument to distinguish between participants who were randomized to the intervention group compared to those who were randomized to the control group. We hypothesized that the intervention group participants, who received access to an enhanced app, would report higher usability scores driven by an increased subscore of usefulness compared to the control group participants.

Criterion validity is the extent to which a measure is correlated with a validated outcome measure [40]. To assess criterion validity, we used the correlation between the PSSUQ subscale and overall scores and the Health-ITUES subscale and overall scores[41].

## Results

A total of 92 persons completed the Health-ITUES survey. Twenty participants completed the survey as part of the end-user usability testing of the beta version of the app and 72 persons completed the survey on enrollment into the 12-week trial. As noted in Table 1, the majority of participants were African American/black and had an annual median household income of less than US $20,000. Nearly all our participants used their mobile phones several times per day. The mean age of the participants was 50.0 (SD 10.5) years with a range of 23.0 to 72.0 years. There was no significant correlation between demographic characteristics, such as income and education, and each of the Health-ITUES subscales.

The range, mean, median, and standard deviation for each of the subscales’ scores at baseline are reported in Table 2. Completion rates were identical for all subscales.

Variability was evaluated for each of the subscales. The full range of responses was not observed for any of the Health-ITUES subscales. Slightly more than a half (59%) scored the maximum score for the quality of life scale, a third (36%) scored the maximum score for the perceived usefulness scale, slightly more than a half (60%) scored the maximum score for the perceived ease of use scale, and more than a third (42%) scored the maximum score for the user control scale. No users scored the lowest possible score. Overall, the users rated the usability of mVIP app as being high. The full range of usability scores was not demonstrated because no users rated the app as the lowest possible score.

By examining the boxplots and the raw data, extremely low scores were treated as outliers and were removed from the reliability and validity analysis. There is evidence that all the subscales were negatively skewed, indicating more favorable usability scores.

### Internal Consistency Reliability and Construct Validity

Internal consistency reliability, as measured by the Cronbach alpha coefficient, is reported for each of the multi-item scales in Table 3. All scales displayed very good Cronbach alpha values (>.7) with the scores ranging from .85 to .92. All the Cronbach alpha values were less than .95, which indicates no redundancy in items [32]. Interscale correlations were all less than the corresponding Cronbach alpha values and ranged from .56 to .82, which indicates moderate to strong correlations [42]. Impact was more correlated with perceived usefulness than with the perceived ease of use and user control (*r*=.82, *r*=.63, and *r*=.56, respectively), which is evidence of convergent validity and discriminant validity.

Known-groups validity, another subtype of construct validity, was evaluated by measuring differences in mean scale scores at baseline between the control and intervention group (Table 4). As noted previously, the subscale scores were skewed and thus the assumption of normality might be violated for independent samples *t* test, therefore we used a nonparametric method, the Kruskal-Wallis test, to detect differences between the intervention and control group scores. Using the Kruskal-Wallis test, we found a statistically significant difference between the intervention and control group on all subscales expect impact.

### Criterion Validity

Criterion validity was measured through the assessment of the concurrent validity by comparing the measure in question, the Health-ITUES, with an outcome assessed at the same time, the PSSUQ. Table 5 presents information on criterion validity, measured as the correlation between each of the subscales of the Health-ITUES with each of the subscales of a validated usability measurement tool, the PSSUQ. Each of the Health-ITUES subscales and the overall scale were moderately to strongly correlated with the PSSUQ scales. These correlations were significant at the *P*<.001 level. Correlations ranged from .46 to .70. None of the correlations were greater than .80 or were very strong and thus there was no redundancy in subscales [42]. The least correlated subscales of the measures were impact and information quality and the most highly correlated items were the overall scale scores.

**Table 1:** Participant characteristics (N=92).

Characteristics		n (%)
**Gender**		
	Male	50 (54)
	Female	40 (43)
	Transgender (FTM)	1 (1)
	Other	1 (1)
**Race**		
	African American/black	67 (74)
	White	8 (9)
	Other	16 (18)
	Ethnicity	
	Hispanic	19 (21)
	Non-Hispanic	73 (79)
**Sexual orientation**		
	Homosexual/gay/lesbian	24 (33)
	Heterosexual/straight	44 (61)
	Bisexual	3 (4)
	Other	1 (1)
**Education**		
	Elementary	1 (1)
	Some high school	16 (17)
	High school diploma or equivalent	27 (29)
	Some college	28 (30)
	Associate/technical degree	5 (5)
	Bachelor/college degree	15 (16)
**Annual income (US$)**		
	Less than $10,000	41 (45)
	$10,000-$19,999	24 (26)
	$20,000-$39,999	14 (15)
	$40,000-$59,999	1 (1)
	$60,000-$79,999	1 (1)
	$80,000-$99,999	1 (1)
	Don’t know	5 (5)
	Prefer not to answer	5 (5)
**Relationship status**		
	Married or in a steady relationship	22 (31)
	Single, separated, divorced, or widowed	48 (67)
	Other	2 (3)
**Most frequently used mobile devices**		
	Android phone	62 (67)
	iPhone	23 (25)
	Tablet	5 (5)
	Other	2 (2)
**Frequency of using the mobile device**		
	Several times every day	82 (89)
	Once a day	5 (5)
	Several times per week	4 (4)
	Several times per month	1 (1)
**Download/use apps on the mobile device**		
	Yes	80 (88)
	No	12 (13)

**Table 2:** Descriptive statistics: scale scores at enrollment for the Health-ITUES subscales (N=92).

Scale	Mean (SD)	Median (range)	Floor, %	Ceiling, %
Impact	4.5 (0.7)	5.0 (1.3-5.0)	0	59
Perceived usefulness	4.3 (0.8)	4.5 (1.7-5.0)	0	36
Perceived ease of use	4.6 (0.8)	5.0 (1.2-5.0)	0	60
User control	4.2 (0.9)	4.5 (1.7-5.0)	0	42
Overall Health-ITUES score	4.4 (0.7)	4.6 (1.6-5.0)	0	33

**Table 3:** Internal scale consistency scores and interscale correlations for Health-ITUES subscales (N=83).

Scale	Impact		Perceived usefulness		Perceived ease of use			User control		
	Cronbach alpha	*r*	Cronbach alpha	*r*		Cronbach alpha	*r*		Cronbach alpha	*r*
Impact	.85									
Perceived usefulness		.82	.92							
Perceived ease of use		.63		.69		.92				
User control		.56		.68			.61		.86	

**Table 4:** Mean scale scores at baseline by intervention versus control groups for Health-ITUES subscales (N=83).

Scale	Intervention (n=49) Mean (SD)	Control (n=34) Mean (SD)	*P*^a^
Impact	4.70 (0.52)	4.53 (0.61)	.13
Perceived usefulness	4.58 (0.59)	4.28 (0.60)	.01
Perceived ease of use	4.77 (0.48)	4.52 (0.64)	.03
User control	4.45 (0.73)	4.11 (0.82)	.048
Overall Health-ITUES score	4.62 (0.51)	4.35 (0.56)	.01

^a^From Kruskal-Wallis test.

**Table 5:** Correlations between Health-ITUES and PSSUQ subscales (N=83).

PSSUQ	Health-ITUES, *r*^a^				
	Impact	Perceived usefulness	Perceived ease of use	User control	Overall
System usefulness	.63	.53	.57	.49	.63
Information quality	.46	.56	.52	.67	.65
Interface quality	.56	.49	.50	.55	.61
Overall	.60	.60	.59	.64	.70

^a^All correlations significant at the *P*<.001 level.

## Discussion

This paper presents the validation of an instrument for measuring usability of mHealth technology. The validation study reported in this paper was conducted to explore the psychometric properties of the Health-ITUES, a customizable usability evaluation instrument that includes subscales of impact, perceived usefulness, ease of use, and user control in a sample of community-dwelling adults living with HIV who were users of a mobile app for symptom self-management. The Health-ITUES varies from most traditional measurement scales in that it is designed to support customization at the item level to match the specific task/expectation and health IT system while retaining comparability at the construct level. The results of this study support the construct and criterion validity and reliability of the Health-ITUES for usability assessments of mobile technologies.

In this study, the reliabilities of the four Health-ITUES subscales and overall score were examined in terms of internal consistency. The internal consistency reliability of each of the subscales were high but less than the Cronbach alpha=.95 threshold suggesting that there is no indication of redundancy in scale items. Moreover, a comparison of the results from the Health-ITUES to the PSSUQ had moderate to strong correlations (*r*=.46-.70). Information quality, a subscale on the PSSUQ, had the lowest correlation (*r*=.46, *P*<.001), which is to be expected because the constructs are substantively different. The overall scale scores for the PSSUQ and the Health-ITUES had the highest correlation (*r*=.70, *P*<.001). This would be expected because the two usability scales have some overlap in their constructs. At the same time, the overall correlations were only moderately strong [42], suggesting that these two instruments are not redundant and demonstrating good criterion validity.

A strength of this validation is our study sample, which further supports the utility of this instrument for usability assessments for consumer health informatics. Our study sample is comprised of nearly all racial and ethnic minority persons and those persons of the lowest socioeconomic groups in the United States. Nonetheless, our study participants were owners and frequent users of mobile phone devices, demonstrating the widespread use of mobile technology and the relevance of mHealth technology across end users. In contrast to the earlier development and validations of the Health-ITUES in nurses using a Web-based system [25,26], this paper presents a validation of this instrument for mHealth technology and with patients [43].

Construct validity was assessed by measuring differences in mean scale scores at baseline between control group and intervention group participants. Statistically significant differences between groups were found in the expected direction for all subscales except impact. These trends were expected because at baseline we would not expect to find a significant difference in impact as a result of the study intervention. In contrast, a statistically significant difference in perceived usefulness, ease of use, and user control was demonstrated, with intervention group participants finding the mobile app to be more usable than control group participants did.

The construct validity of the measure was also supported by the degree to which interscale correlations corresponded to what was expected. The higher correlations among the impact and perceived usefulness subscales than between the other subscale items supports the convergent and discriminant validity of this instrument because participants who perceive the technology to be useful are more likely to show an improvement in their impact as a response.

There are a number of limitations of this validation study. First, most of our study sample were mobile phone users and so we were unable to validate the use of this instrument as a usability assessment tool in non-mobile phone users. This limitation is mitigated as the number of mobile phone users continues to grow across geographic regions and socioeconomic groups. A second limitation is that these findings may not be generalizable beyond our study population. Further research is most definitely needed to further validate this instrument in other study populations. Given the dearth of current instruments for usability assessments, we believe that the findings from this study provide an important contribution to the literature and an opportunity for future study of this instrument with additional study populations.

This study provides preliminary evidence to support the validity and reliability of the Health-ITUES for usability assessments of mobile technology. In light of our findings, the authors recommend use of the Health-ITUES as a measurement tool for assessing the usability of mobile technologies. Validation of the Health-ITUES is an important step in ensuring that usability is attended to and maintained in mHealth technology interventions. This work is particularly important given the proliferation of mobile technologies and the push for consumers to take control of their own health and become greater users of technology for the acquisition and delivery of health information. Further, these findings will hopefully stimulate additional research and practice to ensure the usability of mobile technologies and particularly for those tools that are developed for consumers’ use.

## Abbreviations

AIDS: acquired immune deficiency syndrome
Health-ITUEM: Health Information Technology Usability Evaluation Model
Health-ITUES: Health Information Technology Usability Evaluation Scale
IT: information technology
mHealth: mobile health
PSSUQ: Post-Study System Usability Questionnaire

## References

[1] We Are Social.

[2] Catalani C, Philbrick W, Fraser H, Mechael P, Israelski DM (2013). mHealth for HIV treatment & prevention: a systematic review of the literature. Open AIDS J.

[3] Devi BR, Syed-Abdul S, Kumar A, Iqbal U, Nguyen P, Li YJ, Jian W (2015). mHealth: an updated systematic review with a focus on HIV/AIDS and tuberculosis long term management using mobile phones. Comput Methods Programs Biomed.

[4] Eysenbach G, Wyatt J (2002). Using the Internet for surveys and health research. J Med Internet Res.

[5] Wyatt JC (2000). When to use web-based surveys. J Am Med Inform Assoc.

[6] IMS Institute for Healthcare Informatics.

[7] Iribarren S, Schnall R (2017). Health Informatics: An Interprofessional Approach.

[8] Schnall R, Cho H, Webel A (2017). Predictors of willingness to use a smartphone for research in underserved persons living with HIV. Int J Med Inform.

[9] Schnall R, Bakken S, Rojas M, Travers J, Carballo-Dieguez A (2015). mHealth technology as a persuasive tool for treatment, care and management of persons living with HIV. AIDS Behav.

[10] (2011). mHealth: New horizons for Health Through Mobile Technologies: Second Global Survey on eHealth.

[11] Furlow B (2012). Clinical Advisor.

[12] Zapata B, Fernández-Alemán JL, Idri A, Toval A (2015). Empirical studies on usability of mHealth apps: a systematic literature review. J Med Syst.

[13] Holzinger A (2005). Usability engineering methods for software developers. Commun ACM.

[14] Schnall R, Rojas M, Travers J, Brown W, Bakken S (2014). Use of design science for informing the development of a mobile app for persons living with HIV. AMIA Annu Symp Proc.

[15] Schnall R, Rojas M, Bakken S, Brown W, Carballo-Dieguez A, Carry M, Gelaude D, Mosley JP, Travers J (2016). A user-centered model for designing consumer mobile health (mHealth) applications (apps). J Biomed Inform.

[16] Sheehan B, Lee Y, Rodriguez M, Tiase V, Schnall R (2012). A comparison of usability factors of four mobile devices for accessing healthcare information by adolescents. Appl Clin Inform.

[17] Maguire M (2001). Methods to support human-centred design. Int J Hum-Comput St.

[18] Georgsson M, Staggers N (2016). Quantifying usability: an evaluation of a diabetes mHealth system on effectiveness, efficiency, and satisfaction metrics with associated user characteristics. J Am Med Inform Assoc.

[19] Brown W, Yen PY, Rojas M, Schnall R (2013). Assessment of the Health IT Usability Evaluation Model (Health-ITUEM) for evaluating mobile health (mHealth) technology. J Biomed Inform.

[20] Yen P, Bakken S (2012). Review of health information technology usability study methodologies. J Am Med Inform Assoc.

[21] Davis F, Bagozzi R, Warshaw P (1989). User acceptance of computer technology: a comparison of two theoretical models. Manage Sci.

[22] International Organization for Standardization.

[23] Househ MS, Shubair MM, Yunus F, Jamal A, Aldossari B (2015). The Use of an Adapted Health IT Usability Evaluation Model (Health-ITUEM) for evaluating consumer reported Ratings of Diabetes mHealth Applications: Implications for Diabetes Care and Management. Acta Inform Med.

[24] Yasini M, Beranger J, Desmarais P, Perez L, Marchand G (2016). mHealth Quality: a process to seal the qualified mobile health apps. Stud Health Technol Inform.

[25] Yen P, Wantland D, Bakken S (2010). Development of a customizable health IT usability evaluation scale. AMIA Annu Symp Proc.

[26] Yen P, Sousa K, Bakken S (2014). Examining construct and predictive validity of the Health-IT Usability Evaluation Scale: confirmatory factor analysis and structural equation modeling results. J Am Med Inform Assoc.

[27] Folstein MF, Folstein SE, McHugh PR (1975). “Mini-mental state”. A practical method for grading the cognitive state of patients for the clinician. J Psychiatr Res.

[28] Lewis J (2002). Psychometric evaluation of the PSSUQ using data from five years of usability studies. Int J Hum-Comput Int.

[29] Lewis J (1992). Psychometric evaluation of the post-study system usability questionnaire: the PSSUQ. Proceedings of the Human Factors and Ergonomics Society Annual Meeting.

[30] Lewis J, Henry S, Mack R (1990). Integrated office software benchmarks: a case study. Proceedings of the third IFIP Conference on Human-Computer Interaction–INTERACT.

[31] Sauro J, Lewis J (2016). Quantifying the User Experience: Practical Statistics for User Research.

[32] Nunnally J, Bernstein IH (1994). Psychometric Theory.

[33] Cronbach L (1951). Coefficient alpha and the internal structure of tests. Psychometrika.

[34] Tavakol M, Dennick R (2011). Making sense of Cronbach's alpha. Int J Med Educ.

[35] National Council on Measurement in Education.

[36] Brains C (2011). Empirical Political Analysis.

[37] Cronbach L, Meehl P (1955). Construct validity in psychological tests. Psychol Bull.

[38] Campbell DT, Fiske D (1959). Convergent and discriminant validation by the multitrait-multimethod matrix. Psychol Bull.

[39] National Survey of Student Engagement.

[40] (1985). Standards for Educational and Psychological Testing.

[41] Justice A, Holmes W, Gifford A, Rabeneck L, Zackin R, Sinclair G (2001). Development and validation of a self-completed HIV symptom index. J Clin Epidemiol.

[42] Evans J (1996). Straightforward Statistics for the Behavioral Sciences.

[43] Deeks S, Lewin S, Havlir D (2013). The end of AIDS: HIV infection as a chronic disease. Lancet.

